# Fifty Years and Counting: Searching for the “Silver Bullet” or the “Silver Shotgun” to Mitigate Preharvest Aflatoxin Contamination

**DOI:** 10.3390/toxins17120596

**Published:** 2025-12-15

**Authors:** Baozhu Guo, Idrice Carther Kue Foka, Dongliang Wu, Josh P. Clevenger, Rong Di, Jake C. Fountain

**Affiliations:** 1 Crop Genetics and Breeding Research Unit, USDA-Agricultural Research Service, Tifton, GA 31793, USA; 2 Department of Plant Biology, Rutgers, The State University of New Jersey, New Brunswick, NJ 08901, USA; ik351@sebs.rutgers.edu; 3 Department of Plant Pathology, University of Georgia, Tifton, GA 31793, USA; dongliang.wu@uga.edu; 4 HudsonAlpha Institute for Biotechnology, Huntsville, AL 35806, USA; jclevenger@hudsonalpha.org; 5 Department of Plant Pathology, University of Georgia, Griffin, GA 30223, USA

**Keywords:** preharvest aflatoxin contamination, “living embryo” theory, “Key Largo” theory, host resistance, drought stress, oxidative stress, reactive oxygen species, ROS, CRISPRa, tissue culture, enhanced immunity, food safety

## Abstract

The year 2025 marks two significant milestones for aflatoxin research: 65 years since aflatoxin was first identified in 1960, and 50 years of focused research on preharvest aflatoxin contamination since it was first recognized in 1975. Studies in the 1970s revealed that *A. flavus* could infect crops like maize and produce aflatoxin in the field before harvest and made it possible to investigate the potential genetic resistance in crops to mitigate the issues. Tremendous efforts have been made to learn about the process and regulation of aflatoxin production along with interactions between *A. flavus* and host plants as influenced by environmental factors. This has allowed for the breeding of more resistant crops and investigations into the underlying genetic and genomic components of resistance mechanisms in crops like maize and peanut. However, despite decades of studies, many questions remain. One established “dogma” is that drought stress, especially when combined with high temperatures, is the single greatest contributing factor to preharvest aflatoxin contamination and is a perennial risk faced throughout the major agricultural production regions of the world. Although there are many reviews summarizing the decades’ long wealth of information about *A. flavus*, aflatoxin biosynthesis, management and host plant resistance, there are few reports that put the spotlight on why aflatoxin contamination is exacerbated by drought stress, which places plants under severe physiological stress and weakens immune systems. Therefore, here we will focus on three major areas of research in maize: the “living embryo” theory and host resistance mechanisms, the “Key Largo hypothesis” and the causes of drought-exacerbated aflatoxin contamination, and recent advancements in CRISPR-based genome editing for enhancing drought tolerance and increasing plant immune responses. This will highlight key breakthroughs and future prospects for the continuing development of superior crop germplasm and cultivars and for mitigating aflatoxin contamination in food and feed supply chains.

## 1. Introduction

*Aspergillus flavus* is a ubiquitous saprophytic and opportunistic fungal pathogen infecting, colonizing, and producing aflatoxin in agricultural commodities worldwide [1]. *A. flavus* was described as early as 1809, but the initial discovery of aflatoxin stemmed from the “Turkey X disease” outbreak in England in 1960 [2,3], which was a new, highly fatal disease that struck flocks of young turkeys in England. The disease was characterized by exceptionally high mortality in infected flocks. The cause of the disease remained a mystery for a while, leading to the designation “Turkey X disease”. The subsequent identification of the toxin produced by *A. flavus* sparked a new era of aflatoxin research, one of the most potent natural carcinogens ever discovered and causing a range of health issues, including liver damage, immune suppression, and stunted growth in children [4].

Aflatoxin contamination was treated as a post-harvest storage problem until Anderson et al. (1975) reported an extensive 3-year field survey, covering all the corn-producing areas of the United States and sampling from 6 weeks prior to harvest through harvest, and supported pre-harvest aflatoxin contamination occurring by the late milk stage, and the highest rates of incidence were in the warmer and humid growing regions like southern states [5,6]. This 1975 milestone shifted mitigation strategies to intervene before harvest and made it possible to investigate crop-plant genetic resistance and to develop crops that could resist fungal infection and aflatoxin production in the field before harvest. Scully et al. (2008) reported a long-term survey, starting shortly after 1975, covering 45 Georgia counties to monitor the regional severity of aflatoxin contamination in corn before harvest for 28 years [7]. The findings were alarming, with an average of 97 ppb through the 28-year survey, well above the 20 ppb FDA limit [7]. The realization of the urgency and the unique nature of the aflatoxin problem and the need for novel technologies to ameliorate the impact of food safety became a focal point of discussion at the first U.S. Aflatoxin Elimination Workshop held in New Orleans, LA, in 1988. This workshop was held annually as an international forum to assemble scientists and representatives of the different commodities (corn, peanut, tree nuts, and cotton) and industries in a unique cooperative effort to develop aflatoxin control strategies through research and development [8,9]. Since the 1970s, with the dramatically shifted paradigm of managing *A. flavus* and aflatoxin contamination from passive post-harvest prevention to active breeding of resistant crops to fungal infection, research efforts have been focusing on determining the source of host plant resistance to prevent *A. flavus* colonization and subsequent aflatoxin production before harvest [7]. These efforts have employed numerous techniques and approaches, including modern plant breeding and genomic tools such as proteomic, transcriptomic, and biochemical analyses to discover the underlying mechanism of host plant resistance and the interaction between the host crops and the fungus [10]. To date, these efforts have revealed that resistance is quantitatively inherited with a strong genotype-by-environment component [11,12].

In the United States, the Southeastern Regional Aflatoxin Trial (SERAT) was formed under the umbrella of the U.S. Aflatoxin Elimination Workshop in 2003, and its mission is to test and identify public germplasms, inbreds, and hybrids with the most consistent resistance to aflatoxin accumulation and to evaluate their essential agronomic traits in different environments [8,9]. SERAT has been testing 30 to 40 public breeding hybrids for agronomics and aflatoxin accumulation each year since 2003 [13]. The SERAT trials demonstrated that a large portion of aflatoxin susceptibility is genetic (22%; for yield this was 19%) and heritable across very diverse but relevant environments [13,14].

Nonetheless, at this time, there is no “Silver Bullet” having been found for solving the aflatoxin contamination problem in crops before and after harvest, and the “Silver Shotgun” of all available strategies must be applied simultaneously at all stages in the supply chain to ensure a healthy crop, free of aflatoxins [12,15,16]. The most explored method for mitigating aflatoxin contamination is developing and using preharvest host resistance, because *A. flavus* infects and produces aflatoxins in susceptible crops prior to harvest. Another factor, the well-established “dogma”, is that drought stress plays a crucial and multi-faceted role in both promoting and suppressing host immune responses against various abiotic and biotic stresses, resulting in exacerbated aflatoxin accumulation [17,18]. It is a complex interaction with a high degree of environmentally induced variability, with abiotic and biotic stress strongly influencing resistance or susceptibility. As early as in 1993, Brown et al. (1993) reported a “living embryo” hypothesis and suggested that a living embryo’s metabolic activity in corn is crucial for maintaining the resistance and reducing aflatoxin contamination, as the resistance is lost in “non-living” damaged embryos [19]. Understanding how the viable embryo mediates resistance to aflatoxin accumulation may provide opportunities to modify corn genotypes to support lower levels of contamination, and the “living embryo” theory offers the basis for studies of the resistance mechanism of corn plants [20] in contrast to the newly reported “Key Largo” hypothesis related to investigating the potential role of reactive oxygen species (ROS) in host-drought-*A. flavus* interactions [18,21]. The success in the advancement of genome-editing, like genome-wide knockout and activation as powerful platforms, will play significant roles in research mitigating strategies of aflatoxin contamination. Therefore, this review will focus on three areas of research in maize: the “living embryo” theory and the associated host resistance mechanisms, the “Key Largo” theory and the causes of aflatoxin contamination exacerbated by drought stress, and lastly, leveraging advancements in CRISPR (clustered regularly interspaced short palindromic repeats)-based genome editing, particularly the integration of CRISPR with tissue culture techniques for enhancing drought tolerance and reducing aflatoxin contamination, highlighting key breakthroughs, challenges, and future prospects.

Because of safety concerns of the toxicological content in food and feed, it is an urgent need to search the mitigation strategies to reduce the level or eliminate the contamination in food chain. Aflatoxin B_1_ is classified by the International Agency for Research on Cancer (IARC) as a Group 1 human carcinogen and is strongly associated with hepatocellular carcinoma and other adverse health outcomes [22,23]. Consequently, mitigation success must be defined by the ability to meet the maximum residue limits (MRLs) such as the U.S. FDA limit of 20 ppb for food and feed and more stricter European Union limits of 2–4 ppb for human consumption [24], and Codex Alimentarius guidelines for contaminants in food and feed [25]. Therefore, this review puts the spotlight on why aflatoxin contamination is exacerbated by drought stress and the causes of drought-exacerbated aflatoxin contamination to safeguard food and feed supply chains.

## 2. The “Living Embryo” Theory and Maize Host Resistance Mechanisms, a Case Study of GT-MAS:gk

Broad genetic variation regarding host resistance to aflatoxin accumulation exists across both maize and peanut germplasm. In maize, kernel infection assays and multi-environment trials of diverse inbred lines demonstrate substantial natural resistance, though none exhibit full immunity. Since Zuber et al. (1978) proved resistance to aflatoxin accumulation is a heritable trait in maize [26], many resistant breeding lines have been developed and released. Among the reported resistant germplasm, a maize germplasm GT-MAS:gk was developed and released as resistant to aflatoxin accumulation in Georgia [19,20]. However, Guo et al. (2001) demonstrated the resistance in GT-MAS:gk was still segregating [27], and inbreeding selection was made and tested in the field. Later three inbred lines, GT601, GT602, and GT603 [28,29], were released for public use.

In 1993, Brown et al. first tested GT-MAS:gk and proposed the “living embryo” theory that metabolic activity within the living corn embryo produces compounds that are key to the corn kernel’s resistance to aflatoxin contamination [19], which laid the groundwork for study of causal genes, proteins, and metabolites [30]. This theory is supported by evidence that resistance diminishes when the embryo is killed, wounded, or removed, as reported by Guo (1995), who investigated the mechanisms of resistance to aflatoxin accumulation by *A. flavus* in maize genotype GT-MAS:gk [31].

Guo et al. (1995) first reported that genotype differences in aflatoxin B_1_ accumulation were demonstrated between GT-MAS:gk and thirteen maize hybrids [20]. Most commercial hybrids supported high levels of aflatoxin production, with no differences in aflatoxin levels between intact and wounded kernels of these genotypes. In addition, aflatoxin levels were higher in wounded than in intact kernels. GT-MAS:gk not only supported the lowest levels of aflatoxin production in intact kernels, but aflatoxin levels in endosperm-wounded kernels were also significantly lower in GT-MAS:gk than in wounded kernels of all other tested lines. Furthermore, treatment with KOH to remove cutin from intact kernels prior to inoculation with *A. flavus* caused a substantial increase in aflatoxin accumulation in GT-MAS:gk, whereas only marginal increases were seen in the susceptible hybrid Pioneer 3154. Similarly, removing wax from the surface of GT-MAS:gk kernels greatly increased their susceptibility to aflatoxin accumulation. Interestingly, when wax removal was combined with treatment with KOH or purified cutinase, aflatoxin levels in kernels were equal to those in wounded control kernels in both genotypes. These results indicated that wax and cutin layers of maize kernel pericarps play a critical role in resistance to aflatoxin accumulation in GT-MAS:gk. In addition, detailed studies of corn kernel wax and fungal cutinase production by *A. flavus* [32] revealed that GT-MAS:gk possesses a greater quantity and unique composition of kernel wax, which may serve as a barrier to *A. flavus* infection [33]. Although cutinase produced by *A. flavus* appears to facilitate active infection of corn kernels [32]. *A. flavus* secretes extracellular cutinase when growing on cutin-containing medium, suggesting *A. flavus* acts as an opportunistic pathogen utilizing cutinase as a virulent factor for establishing infection under field conditions. To evaluate the potential role of kernel wax as a physical barrier to *A. flavus* infection and colonization, Russin et al. (1997) dissolved wax from various maize genotypes in chloroform or acetone and mixed it with autoclaved A and M medium [33]. Remarkably, only wax extracts from GT-MAS:gk exhibited antifungal activity. Thin-layer chromatography further revealed a unique compound present exclusively in GT-MAS:gk but also lacking a peak common to all others.

Another interesting phenomenon related to kernel germination or sprouting was observed when kernels of both resistant and susceptible genotypes were preincubated for three days at 100% relative humidity (RH) prior to inoculation with *A. flavus*; germination levels increased dramatically compared to kernels that were not preincubated [34]. In preincubated kernels aflatoxin levels remained consistently low in GT-MAS:gk but decreased markedly (61%) in Pioneer 3154. When eight susceptible hybrids were evaluated for aflatoxin accumulation under the same preincubation conditions, seven exhibited significantly lower toxin levels compared to kernels that were not preincubated. On average, aflatoxin reduction across all tests was 83%, ranging from 68% to 96% [34]. This preincubation-induced germination, or “physiological sprouting”, was associated with a reduction in aflatoxin accumulation, which might be attributed to the induction of specific antifungal proteins, such as zeamatin and ribosome-inactivating protein (RIP) [35]. The distributions of these two specific antifungal proteins in maize kernel tissues indicate that RIP-like protein was present at higher levels within endosperm than in embryo tissues, whereas zeamatin-like protein was more concentrated in embryo than in endosperm [36].

The “living embryo” theory further suggests that resistant corn kernels lose their resistance to *A. flavus* and subsequent aflatoxin accumulation when the embryo is damaged or inactivated, pointing to an active, metabolic defense system involving the production of specific proteins and other protective metabolic compounds. Research has identified several resistance-associated proteins (RAPs) that contribute to the resistance observed in corn. In summary, there appear to be two primary levels of disease resistance: one at the pericarp, where the pericarp waxes play a critical physical role in preventing infection [20], and another at the sub-pericarp level, where RAPs function as biochemical defenses against *A. flavus* colonization [30,37,38]. Several RAPs, especially those constitutively expressed in corn kernels, have been identified as the second layer of defense. These include maize RIP and zeamatin that concentrate in the aleurone layer to protect the endosperm and embryo, respectively [36], in addition to storage proteins such as globulin 1 (GLB1) [39]; stress-responsive proteins such as glyoxalase 1 (GLX1) [40]; antifungal proteins such as the 14 kDa trypsin inhibitor protein (TI); pathogenesis-related protein 10 (PR10) [41]; and a novel β-1,3-glucanase [42].

The germination-induced resistance as reported by Guo et al. [34] suggests that the process of germination, a metabolic activity of the living embryo, activates defense mechanisms [34]. This finding aligns with reports that *A. flavus*-resistant maize tends to accumulate higher antioxidant enzymes, such as peroxidase, and exhibit greater tolerance to drought and heat stress compared to susceptible varieties. This phenomenon may be explained by the findings of Hite et al. (1999), who demonstrated an inverse correlation between catalase activity and hydrogen peroxide (H_2_O_2_) levels in maize during seed germination [43]. In all examined maize lines, H_2_O_2_ levels were highest during the early 1–2 days post-imbibition (or preincubation) and decreased thereafter, but the total antioxidant enzyme catalase activity was lowest during the early 1–2 days post-imbibition and reached maximal activity at 4–6 days post-imbibition. Given the fact that oxidative stress caused by increased reactive oxygen species (ROS) such as H_2_O_2_ accumulates to maximum quantities at the first two days post-imbibition followed by increased antioxidative catalase activity beginning at three days in maize kernels [43], a combination of host-derived resistance and antioxidant proteins and reduced ROS production at the time of inoculation may have contributed to the reduction in aflatoxin production [34], which also leads to the next section, “Key Largo” theory [18], to investigate the potential role of drought stress, particularly oxidative stress caused by excessive ROS in host-drought-*A. flavus* interactions, the role of ROS in aflatoxin production regulation, how ROS may function in signaling between host plants and *A. flavus* during infections under drought conditions and their effects on aflatoxin contamination.

Together, these findings clarify two complementary layers of resistance—physical pericarp barriers and embryo-driven biochemical defenses—and provide the biological foundation for Section 3, which examines how drought and oxidative stress reshape host–pathogen interactions.

## 3. The “Key Largo” Theory, Host—Drought—*A. flavus* Interactions and Oxidative Stress

A large body of host–pathogen studies shows that drought and heat elevate oxidative and nitrosative stress in maize, and genotypes that maintain stronger antioxidant capacity tend to be less prone to aflatoxin contamination [44]. A coordinated series of studies from Yang et al. [30,44] further elucidated the tissue-specific redox responses to drought stress. Proteomic analyses of maize kernels revealed broad stress-signaling changes under drought [30]; histochemical and quantitative assays showed that drought-sensitive lines accumulate higher ROS and reactive nitrogen species (RNS) in leaves and developing kernels than drought-tolerant lines, and kernel metabolomics mapped drought-induced shifts in antioxidant and stress-metabolite pathways, with sensitive lines mounting more intense responses consistent with susceptibility patterns [44].

On the fungal side, oxidative stress functions as a key prerequisite for aflatoxin biosynthesis. In *A. parasiticus*, the transition from trophophase to idiophase in toxigenic strains is characterized by increased oxygen demand and enhanced antioxidant defenses precisely as aflatoxin production begins [45]. Complementarily, mutants that stall at successive precursor steps show a graded increase in ROS relative to a nontoxigenic strain, and modest exogenous H_2_O_2_ further elevates AFB_1_ production—linking redox load to pathway induction [46]. Together, these findings position ROS both as a trigger for pathway activation and as a condition to which aflatoxin (and its precursors) contributes adaptive redox homeostasis.

More direct evidence for redox regulation of aflatoxin biosynthesis arose from pathway genetics. In *A. parasiticus*, a comparison of wild type (SU-1) and an *aflR*-disruptant (AFS10) showed that total ROS declines between 24 and 48 h in SU-1 coincident with peak aflatoxin accumulation, whereas AFS10 does not show the same reduction. Notably, exogenous aflatoxin treatment lowers ROS in AFS10 at 24 h without inducing superoxide dismutase (SOD) transcripts, indicating the presence of both *aflR*-dependent and toxin-dependent ROS mitigation mechanisms [47]. Finotti et al. (2021) also showed that aflatoxin B1 can actively scavenge H_2_O_2_ in direct reactions and provide antioxidant protection to *E. coli* when exogenously supplied, providing further evidence for the role of aflatoxins in oxidative stress alleviation [48]. In *A. flavus*, oxidative signals co-regulate development and secondary metabolism, antioxidants that modulate ROS and thiol redox balance suppress sclerotial differentiation, and aflatoxin B1 (AFB1) production tracks with oxidative status [49]. A *veA* deletion mutant lacks both sclerotia and AFB1, underscoring the redox-sensitive developmental control of the pathway [21].

Population-level comparisons reinforce the functional link between aflatoxin capacity and oxidative-stress tolerance. Across highly aflatoxigenic, moderate, commercial atoxigenic, and naturally atoxigenic *A. flavus* isolates, conidia from high-AF producers survive higher H_2_O_2_ than low/atoxigenic isolates, consistent with aflatoxin production (or tightly linked mechanisms) buffering oxidative stress. Complementary transcriptomics revealed oxidative challenges to coordinate changes in development regulators, antioxidant systems, and secondary-metabolism genes [50].

In summary, drought and heat elevate host ROS and RNS, while the fungus senses and integrates oxidative signals to drive development and aflatoxin biosynthesis. Recently, Fountain et al. (2025) summarized this phenomenon, coined the “Key Largo” theory, and described contrasting field scenarios: under non-stress conditions—reminiscent of “sitting on the beach in Key Largo”—with adequate irrigation and optimal temperatures, aflatoxin accumulation remains low due to lack of physiological stress on both host crops and the infecting pathogen *A. flavus* [18]. However, under stressful environmental conditions, particularly drought and high temperatures, aflatoxin contamination tends to increase due to elevated physiological stress in both the host and the fungus. This hypothesis is based on observed responses in sensitive hosts, where elevated ROS under drought and heat stress are perceived by infecting *A. flavus*, leading to exacerbated aflatoxin production. Alternatively, reduced ROS accumulation in drought-tolerant hosts, either under stressful conditions or normal growth, can lead to normal or even reduced aflatoxin contamination. The differences in overall oxidative stress tolerance seen among strains of *A. flavus* with contrasting aflatoxin production capabilities also point to the possible role of aflatoxin production in alleviating oxidative stress in *A. flavus*, with aflatoxin synthesis being further exacerbated by host- or environment-derived oxidative stress.

To test the “Key Largo” theory, a series of experiments examining the responses of various isolates of *A. flavus* with differing levels of oxidative stress tolerance and aflatoxin production capability to H_2_O_2_-derived oxidative stress in vitro was conducted using several different approaches, including transcriptomics [50,51], proteomics [52], and metabolomics [53]. These studies have shed light on isolate-specific responses to oxidative stress correlated with their aflatoxin production levels and identified additional secondary metabolite pathways stimulated by oxidative stresses. Highly aflatoxigenic isolates such as AF13 showed less vigorous responses to stress, while moderate aflatoxin producers such as NRRL3357 and atoxigenic isolates such as K54A exhibit stronger oxidative-stress responses than highly aflatoxigenic strains at equivalent time points, and the biological-control strains showed more vigorous responses across all associated omics studies at the examined time points. Therefore, the role of aflatoxin production as a stress-alleviating mechanism also factors into the overall “Key Largo” theory, as highly aflatoxigenic isolates exhibit a greater level of tolerance to oxidative stress than less aflatoxigenic or non-aflatoxigenic isolates due to more aflatoxin biosynthesis-associated oxidase activity leading to more secondary ROS bursts and subsequent increases in antioxidant expression [54].

These host–pathogen interactions occur within environmental contexts defined by seasonal drought severity, soil-moisture deficits, canopy temperature, and nighttime humidity. Historical records from the southeastern United States show that high-aflatoxin years consistently align with mid-season moisture stress followed by late-season heat [55,56], reinforcing the environmental foundation of the Key Largo hypothesis.

While the primary focus is aflatoxin, it is important to consider whether altering drought-response or antioxidant pathways may inadvertently influence the accumulation of other mycotoxins such as fumonisins or DON [57,58,59]. Comparisons of resistant vs. susceptible germplasm will be required to ensure that preharvest aflatoxin mitigation does not shift risk toward other toxins.

## 4. Integrating CRISPR-Based Genome Editing and Tissue Culture for Drought Tolerance and Aflatoxin Resistance in Crops

To complement the historical and mechanistic perspectives described above, recent advances in CRISPR-based genome editing allow direct manipulation of pathways central to both the living embryo and Key Largo frameworks. These tools provide a molecular bridge from classical resistance mechanisms to modern crop improvement strategies.

Genome-wide activation, knockout, and inhibition are powerful CRISPR-based genome editing approaches to study gene function and identify key regulators of biological processes. Genome-wide activation is a gain-of-function study that uses a modified CRISPR system to upregulate the expression of genes. Genome-wide knockout is a loss-of-function study that ablates gene expression to study the resulting phenotypic changes. Genome-wide inhibition is another loss-of-function study that represses gene expression at the transcriptional level, without altering the underlying DNA sequence. Clevenger et al. (2016) reported identification of a potential susceptibility factor, *ABR1*, as a repressor of ABA signaling that may play a role in permitting preharvest aflatoxin contamination (PAC) [60]. Recent advancements in CRISPR-based genome editing, combined with plant tissue culture techniques, offer transformative solutions. These technologies enable precise genetic modifications, accelerating the development of resilient crop varieties. Given *A. flavus*’ saprophytic nature, an optimal strategy would be to elevate broad immunity within the plant host by enhancing the gene expression of RAPs (resistance-associated proteins) and other effective immunity genes, by suppressing negative regulators of disease resistance such as *2-oxoglutarate Fe(II)-dependent oxygenase* (*2OGO*) [61], and by improving the plant’s fitness under drought and other abiotic stresses. This section explores the integration of CRISPR-mediated genome editing and tissue culture for enhancing drought tolerance and aflatoxin resistance, highlighting key breakthroughs, challenges, and prospects.

The CRISPR/Cas9 system has revolutionized genetic engineering by allowing targeted modifications of plant genomes with unprecedented precision. The system relies on a guide RNA (gRNA) to direct the Cas9 endonuclease to specific genomic loci, inducing double-stranded breaks (DSBs) [62]. These breaks are subsequently repaired via non-homologous end joining (NHEJ), often resulting in gene knockouts, or homology-directed repair (HDR), enabling precise edits. Beyond conventional knockouts, advanced CRISPR variants such as base editing and CRISPR activation (CRISPRa) facilitate single-nucleotide substitutions and transcriptional modulation without DSBs [63]. The groundbreaking and yet relatively underexplored potential of CRISPRa systems offers powerful opportunities for gain-of-function (GOF) studies in plant biology. We advocate for the broader adoption of CRISPRa as a transformative approach to discover and harness genetic variation, ultimately accelerating the development of crops with enhanced disease resistance [64]. These innovations broaden the scope of genome editing, enabling fine-tuned regulation of stress-responsive genes. The CRISPR/Cas9 system has emerged as a powerful and versatile tool for crop genetic improvement, enabling precise genome editing to enhance disease resistance, abiotic stress tolerance, and yield-related traits. Recent advances have demonstrated the potential of CRISPR in peanuts, including targeting *AhMULE9A* to enhance tolerance to aluminum toxicity, a key factor limiting growth on acidic soils [65,66]. In addition, the editing of *AhNFR1* and *AhNFR5* genes via hairy root transformation provided new insights into nodulation mechanisms [67]. Several of the CRISPR targets discussed—such as antioxidant genes, cuticle biosynthesis pathways, and flavonoid regulators—directly intersect the mechanisms highlighted in the living embryo and Key Largo models.

*Tissue culture and transformation methods are central to developing aflatoxin-resistant crops*. Plant tissue culture has long been a cornerstone of agricultural biotechnology, enabling the cultivation of plants under sterile conditions to address goals such as crop improvement, disease resistance, and conservation of germplasm. In addition, it is a crucial step for successful genetic transformation, serving as the regenerative system for delivering CRISPR components and recovering edited plant lines [68]. Core techniques include somatic embryogenesis and organogenesis, which rely on responsive explants such as immature embryos, cotyledons, hypocotyls, or shoot apices [69]. These methods enable dedifferentiation and subsequent regeneration of whole plants from transformed cells, making tissue culture indispensable for crop biotechnology and functional genomics. There are two major approaches widely used for gene delivery: Agrobacterium tumefaciens-mediated transformation and biolistic (particle bombardment) methods. Agrobacterium-mediated transformation is favored for its high efficiency, low transgene copy number, and minimal genomic disruption, making it ideal for stable gene integration in many species [70]. In contrast, biolistic delivery is particularly valuable for crops that are recalcitrant to Agrobacterium, such as certain peanut genotypes or monocots such as maize [68]. Regardless of the delivery method, optimizing tissue culture conditions, including media composition, growth regulators (such as auxins and cytokinins), explant age, and genotype selection is essential for enhancing transformation efficiency and regeneration rates.

When conducting research on aflatoxin mitigation in plants, tissue culture serves as a fundamental component, as it supports various research approaches. For instance, investigating developmental processes under controlled conditions often relies on organogenesis, where maintaining excised roots or shoots in vitro enables researchers to study plant-pathogen interactions during *A. flavus* colonization and evaluate the effects of transgenes or genome edits on organ-specific resistance [71]. Another valuable technique is protoplast culture, which plays a crucial role in functional genomics and gene editing [72]. Isolated protoplasts allow for direct DNA or RNA delivery, supporting transient assays to test candidate genes involved in aflatoxin resistance. Additionally, protoplast fusion enables the combination of genetic material from different species, potentially leading to novel hybrids with enhanced defense traits. Callus culture also provides a versatile platform for studying stress responses, performing genetic transformation, and producing secondary metabolites. In the context of aflatoxin research, callus-based systems facilitate high-throughput screening of maize or peanut lines for resistance-associated traits, such as drought stress tolerance and enhanced antifungal metabolite production.

Therefore, callus induction combined with in vitro screening approaches expands the potential for resistance discovery [73]. One widely used method involves supplementing tissue culture media with polyethylene glycol (PEG) to simulate drought stress, a major predisposing factor for *A. flavus* infection and aflatoxin accumulation. PEG imposes osmotic stress by lowering water potential, enabling researchers to identify calli that maintain growth and regeneration under water-limited conditions [74,75]. Surviving tissues often exhibit physiological and molecular hallmarks of drought tolerance, such as proline accumulation and induction of stress-responsive genes, traits that are tightly linked to reduced aflatoxin contamination in the field.

Hence, advances in tissue culture and transformation not only enable the precise introduction of resistance alleles through CRISPR-based or transgenic strategies but also provide controlled systems for screening stress resilience traits that indirectly mitigate aflatoxin risk. As tissue culture technologies continue to evolve, their integration with in vitro screening methods promises to accelerate the identification, testing, and deployment of resistant germplasm, moving the field closer to a durable “Silver Shotgun” strategy against aflatoxin contamination.

*CRISPR-mediated engineering of drought tolerance*. Drought response in plants is governed by intricate genetic networks regulating osmotic balance, antioxidant responses, calcium efflux, and morphological adaptations such as root architecture and stomatal closure and opening [76,77]. CRISPR gene editing has enabled precise manipulation of these complex pathways, offering a targeted strategy to enhance drought tolerance without compromising growth or yield [78].

Therefore, CRISPR/Cas9 technology has been instrumental in uncovering gene functions and enhancing drought tolerance in major crops. A major focus has been on editing transcription factors such as *DREB*, *NAC*, and *bZIP* families, which act as master regulators of stress-inducible gene expression [78,79]. By activating or repressing downstream genes involved in osmolyte accumulation, membrane stability, and protective protein production, these transcription factors can fine-tune the plant’s response to water deficit. We have recently found success in editing the *AsDREBL* (*Agrotis stolenifera DREB-like*) gene in creeping bentgrass to improve drought tolerance [80]. On the other hand, CRISPRa has demonstrated that manipulating these factors can significantly improve drought resilience in model and crop species like sugarcane [81]. Advances in tissue-specific genome editing have further refined CRISPR applications in plants. In *Arabidopsis*, a highly efficient editing system combining Cas9 and guide RNAs driven by the *AtEF1* promoter enabled precise mutation of *OST2/AHA1*, genes involved in abiotic stress response [82]. This approach yielded new *OST2/AHA1* alleles with improved stomatal regulation and no detectable pleiotropic side effects. These findings demonstrate the potential of spatially controlled gene editing to fine-tune plant responses to environmental stress. Collectively, these studies underscore how CRISPR/Cas9 can be harnessed to enhance agricultural productivity and engineer crops with robust, multi-stress resistance [72].

*CRISPR approaches for aflatoxin resistance*. Aflatoxin contamination is exacerbated by abiotic stresses like drought and high temperatures, which compromise plant defense mechanisms and favor fungal colonization [83]. CRISPR-based gene editing provides a promising avenue for developing aflatoxin-resistant varieties by targeting both host susceptibility factors and fungal infection processes. CRISPR can be used to enhance host resistance by modifying genes that strengthen physical and biochemical defenses. Editing genes associated with pericarp structure and kernel development can improve physical barriers, reducing the likelihood of fungal penetration. For instance, mutations in genes that regulate lipid transfer proteins or cuticle formation may lead to tighter, more resistant seed coats [84]. Also, targeting regulatory enzymes in flavonoid and phenylpropanoid biosynthesis can increase the accumulation of antifungal secondary metabolites [85]. These compounds not only inhibit *A. flavus* growth but may also interfere with aflatoxin biosynthesis [86]. Importantly, reactive oxygen species accumulation under drought stress weakens plant immunity and promotes fungal infection [87,88]. Editing antioxidant-related genes such as those encoding glutathione S-transferases or superoxide dismutases can enhance the plant’s oxidative stress tolerance and indirectly limit aflatoxin accumulation [18]. Further efforts integrating CRISPR with host transcriptomic responses and QTL mapping for aflatoxin resistance could accelerate the identification of new, durable gene targets. A comprehensive, multi-gene editing strategy may ultimately be required to achieve reliable field-level resistance.

*Challenges and future directions*. Despite the significant advancements in CRISPR-based crop improvement, several challenges continue to hinder widespread application. One major obstacle is transformation efficiency, particularly in recalcitrant crops like peanuts, where genotype-dependent tissue culture responses limit the regeneration of edited lines [89,90]. Developing genotype-independent transformation protocols remains a top priority to expand CRISPR accessibility across diverse plant species. Off-target effects also present a concern, potentially leading to unintended mutations that compromise plant fitness or trigger regulatory scrutiny. Advances in guide RNA (gRNA) design algorithms and high-fidelity Cas9 variants have significantly improved targeting specificity, reducing off-target activity and enhancing biosafety [91]. Furthermore, regulatory uncertainty and public acceptance remain key impediments to the deployment of genome-edited crops. While some countries have begun to differentiate gene-edited organisms from traditional GMOs in their regulatory frameworks, global harmonization and transparent communication with stakeholders will be crucial for the responsible commercialization of CRISPR-derived crops [92,93,94].

Emerging technologies offer promising solutions to these challenges. Ribonucleoprotein (RNP) delivery systems, which introduce preassembled Cas9-gRNA complexes directly into plant cells, eliminate foreign DNA integration and reduce regulatory burdens [95]. Likewise, viral vector-mediated CRISPR delivery is gaining traction as a scalable, DNA-free alternative for transient gene editing in planta [96,97]. As these technologies mature, they are expected to broaden the scope and efficiency of CRISPR applications in crop improvement.

## 5. The Path Forward Toward a “Silver Shotgun” Strategy

Since the paradigm shift in the 1970s—from post-harvest prevention to preharvest genetic evaluation and breeding of resistant crops—over 50 years of research have revealed much about genetic resistance and the factors that affect the complex process of host and *A. flavus,* interaction with recent studies revealing the potential ROS-mediated crosstalk between hosts and *A. flavus*. The current data supports an underlying aflatoxin production, which involves *A. flavus* self-protection and survival within a high-ROS environment, including other secondary metabolites like kojic acid. CRISPR is a powerful tool for creating disease-resistant crops, and the integration of CRISPR genome editing and plant tissue culture has opened new frontiers in the development of crops resilient to climate-induced stresses and mycotoxin contamination. Targeted editing of drought-responsive genes and the engineering of host–pathogen resistance mechanisms illustrate the precision and versatility of CRISPR for addressing real-world agricultural challenges. As researchers continue to refine transformation techniques and uncover novel gene targets, these tools will become even more powerful in enabling climate-adapted agriculture. Realizing their full potential will require not only technical advancements but also field validation, regulatory clarity, and proactive engagement with farmers and consumers.

Together, these findings and approaches represent a newly described direction for aflatoxin research. For decades, the research community has sought the proverbial “Silver Bullet”—a single intervention that could reliably and consistently eliminate preharvest aflatoxin contamination. No individual solution is likely to succeed in isolation. Instead, durable progress will require coordinated, multi-component strategies that combine knowledge of host resistance and breeding, fungal biology, and environmental influences with modern tools such as CRISPR genome editing, biotechnology, nanotechnology, and improved forecasting and detection systems. In this context, the success of aflatoxin mitigation must be measured not by percentage reduction alone but by the consistent achievement of aflatoxin concentrations below national and international maximum residue limits (MRLs). Within this broader multi-strategy, atoxigenic *A. flavus* biocontrol remains an important tool; however, their safety must be evaluated not only based on aflatoxin deficiency but also regarding ecological behavior, secondary metabolite profiles, and long-term field persistence. These considerations are increasingly important as biological interventions become more widely adopted and highlight the need for integrated approaches to ensure progress toward a secure, resilient, and aflatoxin-safe food supply.

## Data Availability

No new data were created or analyzed in this study. Data sharing is not applicable to this article.
